# Exploring the PXR ligand binding mechanism with advanced Molecular Dynamics methods

**DOI:** 10.1038/s41598-018-34373-z

**Published:** 2018-11-01

**Authors:** Stefano Motta, Lara Callea, Sara Giani Tagliabue, Laura Bonati

**Affiliations:** Department of Earth and Environmental Sciences, University of Milano-Bicocca, Milan, Italy

## Abstract

The Pregnane X Receptor (PXR) is a ligand-activated transcription factor belonging to the nuclear receptor family. PXR can bind diverse drugs and environmental toxicants with different binding modes, making it an intriguing target for drug discovery. Here we investigated the binding mechanism of the SR12813 ligand to elucidate the significant steps, from the ligand entrance pathway into the binding cavity, to the ligand-induced conformational changes, and to the exploration of its alternative binding geometries. We used the advanced Molecular Dynamics-based methods implemented in the BiKi suite and developed specific methodological approaches to overcome the complexity induced by the buried and flexible binding cavity. The adopted methods provided a full dynamic description of the binding event and allowed rationalization of the observed multiple binding modes. These results suggest that the same approach could be exploited for the study of other binding processes with similar characteristics.

## Introduction

The Pregnane X Receptor (PXR or NR1I2) is a nuclear receptor (NR) that has important roles in drug metabolism and drug-drug interactions. In fact, it regulates the expression of genes encoding drug-metabolizing enzymes (CYPs), which enhancement may lead to an undesired decrease in the bioavailability of many prescribed drugs^1^. For this reason, elucidation of the exact molecular mechanism that underlies PXR activation has important implications for drug development processes^2^. Moreover, PXR has been strongly associated with cancer and with metabolic and inflammatory diseases^1^, making PXR an intriguing new target for drug design studies aimed at developing both antagonist^3^ and agonist molecules^1,2^.

Like the other receptors in the NR family, PXR functions as a ligand-activated transcription factor^2^. Unlike steroid hormone receptors, which are highly selective and work under narrow concentration of their cognate hormone, PXR is an orphan receptor that has evolved to detect several structurally diverse chemicals^4^. Many drugs have been reported to bind PXR, including the antibiotics rifampicin, clotrimazole, ritonavir, but also the antineoplastic drugs cyclophosphamide, taxol and tamoxifen^5^; other ligands are environmental toxicants and dietary constituents^6^. It has been suggested that the human PXR acts as a gene silencer, *i*.*e*. it is constitutively bound to DNA as heterodimer with the Retinoid X receptor (RXR) and in this form it silences transcription of target genes^7^. Ligand binding causes a conformational change leading to the release of co-repressors and the recruitment of co-activators (*e*.*g*. the steroid receptor co-activator, SRC-1)^4^.

Like other NRs, PXR has three types of functional domains: a ligand-binding/dimerization domain; a DNA-binding/weak dimerization domain; and transactivation domains (activation function 1 [AF-1] and 2 [AF-2]). The ligand binding domain (LBD) is located at the C-term of the receptor and forms a heterodimer with RXR^8,9^. The DNA-binding domain is at the N-term and is responsible for recognition of a receptor-specific response element in the promoter region of the target genes^10^. Finally, the transactivation domains consist of a ligand-independent AF-1 domain at the N-term and a C-terminal ligand-dependent transcription AF-2 domain. These domains serve as protein-protein interfaces that guide the recruitment of transcriptional coregulators to the target gene^11^.

The human PXR-LBD structure was crystallized for the first time in 2001^12^ and to date there are 20 depositions in the Protein Data Bank (PDB) of this domain in complex with different ligands. The PXR-LBD is characterized by an “α-helical sandwich”, reproducing the typical NR fold, composed of three layers: α1/α3, α4/α5/α8, and α7/α10 (Supplementary Fig. S1). Unlike in other NRs, in PXR the small β-sheet is expanded to a five-stranded antiparallel β sheet and the α6 helix is often converted to a loop. The latter characteristic is thought to be responsible for the accommodation of different ligands within the internal cavity of the domain^12^. The αAF helix at the C-term of the LBD represents the AF-2 domain involved in binding of co-activators and co-repressors. The interaction with an agonist within the binding cavity leads to the exposure of the hydrophobic surface of αAF and promotes co-activator binding^4^.

All the known crystal structures of PXR complexes include ligands of pharmaceutical interest: the St. John’s wort compound hyperforin^13^; the antibiotic rifampicin^14^; some drug-like ligands^15–17^; the anti-HIV drug PNU-142721^18^. One structure explains the synergistic activation of PXR by the 17α-ethinylestradiol (the active substance of contraceptive pills) and the organochlorine pesticide TNC (an environment contaminant)^19^. Finally, there are four depositions in complex with SR12813^8,12,20,21^, a cholesterol-lowering drug that inhibits cholesterol synthesis increasing degradation of a key reductase^22^. The binding cavity appears buried and with a volume greater than 1,000 Å^3^, noticeably larger than the ones of other NRs^12^. Twenty of the cavity-lining residues are hydrophobic, four are polar and four are charged or potentially charged. A salt-bridge between the E321 and R410 residues effectively neutralizes their charges, so that the inner surface of the cavity is essentially uncharged and hydrophobic^12^. A feature that deserves particular interest for the use of PXR as a drug target is that this promiscuous cavity accepts molecules of widely varying dimensions and chemical properties that occupy different sites with various binding modes. An intriguing case is represented by SR12813, since for this ligand five different orientations inside the cavity have been detected in the experimental structures^8,12,20,21^. It has been suggested that the presence of a protein partner (for example SRC-1^20^, or RXR^8^) could contribute to stabilize a specific binding geometry of the ligand.

The experimental structures of PXR available have provided the basis for several computational investigations that mostly made use of molecular docking methods. These studies were focused on two aims: to find new drugs to either agonize or antagonize the PXR activity^5,23–25^; and to model binding to some environmental pollutants^26–28^. For example, both ligand- and structure-based computational methods have been used to find novel modulators of PXR^5,24^, as well as to screen and predict toxic side-effects of xenobiotics^28^. One of the molecular docking studies suggested that while agonists bind inside the cavity, antagonists bind the AF-2 surface on the exterior of PXR-LBD^23^. However, despite the possibility of using multiple crystal structures for these studies, the molecular docking approach showed several limitations because the protein motions associated to the binding mechanism were not considered^29^. Moreover, the ligand entry or exit pathways to and from the binding cavity are still poorly understood; due to the buried nature of the cavity, there is not an obvious entry or exit route on the surface^28^.

In the recent years, novel methods have been proposed for a full dynamical description of the protein−ligand binding event based on Molecular Dynamics (MD)^30^. Given that the sampling issue is pivotal for the description of these slow processes, enhanced sampling methods are usually employed. Among these Steered MD^31^ and metadynamics^32–34^ were used for the first simulations of drug-binding events, with the ligand moving into and/or out of the binding pocket, and nowadays represent appealing solutions for the drug discovery community.

In this work we focused our attention on the binding mechanism of the most studied PXR ligand, SR12813. We were aimed both at investigating the ligand entrance path into the binding cavity and at elucidating the controversial description of its orientation inside the binding region provided by the multiple experimental crystal structures. The buried nature of the cavity suggests significant conformational rearrangements of the LBD upon ligand entrance. Moreover, the presence of different binding modes implies a high degree of flexibility and plasticity of the domain during the binding process. Therefore, we proposed to explicitly include the dynamic description of the binding event using recent MD-based tools implemented in the BiKi suite^35^. In particular, we employed the MD-Binding method^36^ to analyse the binding mechanism and gain insights into the ligand entrance pathway. Moreover, we proposed the use of the scaled MD (SMD) approach^37,38^ to extensively sample the conformational space available to the PXR-ligand complex, thus allowing elucidation of the SR12813 dynamic behaviour within the binding cavity. The characteristics of the system required the development of specific methodological approaches that may be insightful also for investigation of other ligand binding processes.

## Results

### Evaluation of the experimental binding modes

**Figure 1:**
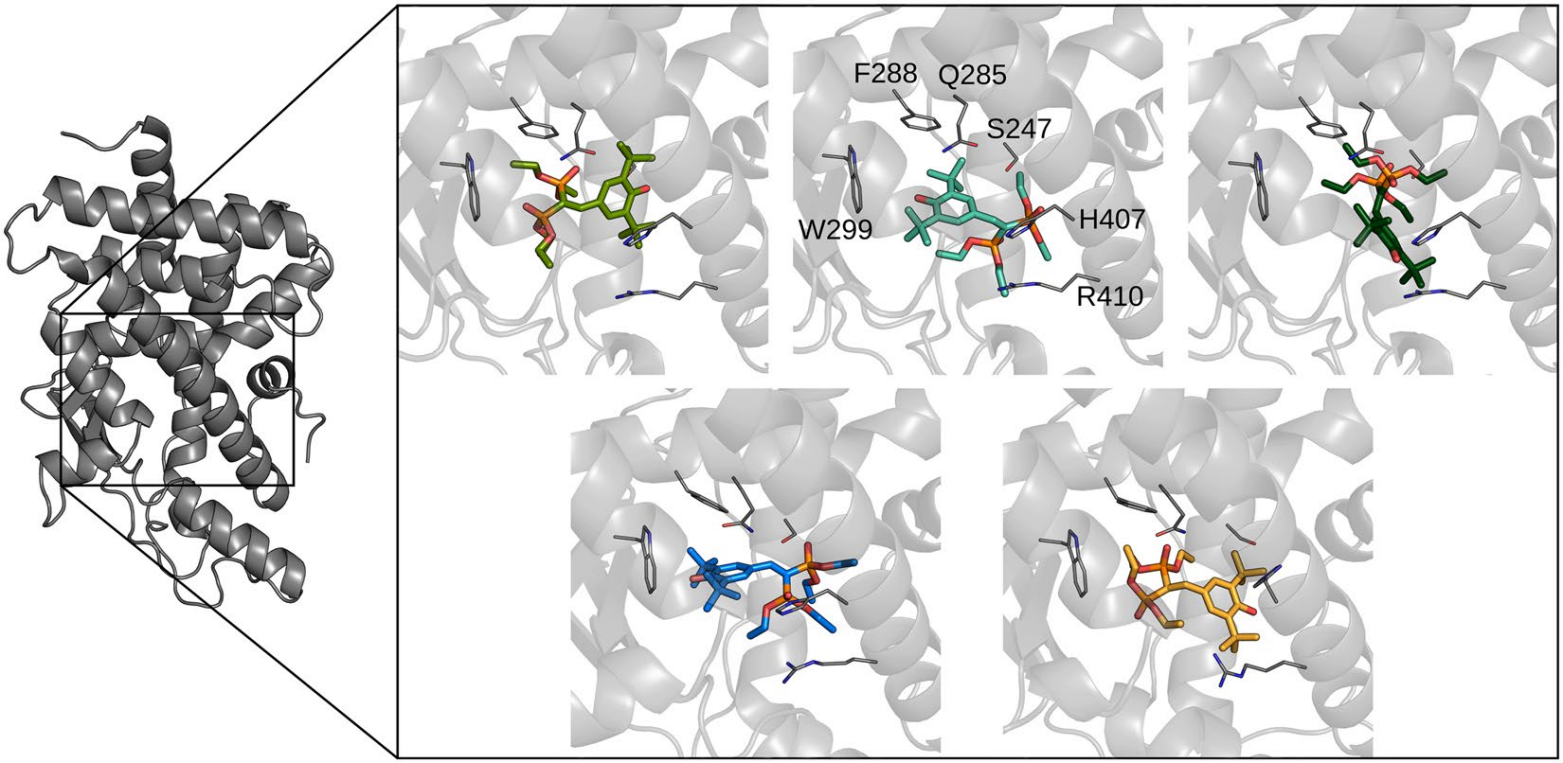
Different structures of the PXR ligand binding domain bound to SR12813. The 1ILH structures^12^, that represent three different ligand binding modes, are reported on top in different shades of green: from left to right, 1ILH.a, 1ILH.b, and 1ILH.c. The 1NRL structure^20^ is reported in blue and the 4J5X structure^8^ in orange. Residues relevant to ligand binding are represented with solid lines and labelled in the 1ILH.b structure.

Among the X-ray structures for the PXR-LBD in complex with SR12813, 1NRL and 3HVL include SRC-1, 4J5X both SRC-1 and RXR, while 1ILH does not present any co-crystallized partners (Supplementary Table S1). The two protein partners bind in different regions: RXR dimerizes with the α9/α10 PXR helices, while SRC-1 binds to the αAF helix. In these structures different binding modes were observed for the ligand (Fig. 1). The 1ILH deposition^12^ represents three different ligand binding modes here named 1ILH.a, 1ILH.b, and 1ILH.c.

Overall the residues within the cavity of the different depositions lie in similar geometries (pairwise RMSD on binding site heavy atoms: 1ILH-1NRL = 1.10 Å, 1ILH-4J5X = 1.40 Å, 1NRL-4J5X = 1.23 Å), with the ligand contacting the same set of residues, but with different orientations. In both the 1ILH.a and 4J5X structures the ligand phosphate groups are directed toward the W299 residue, while the hydroxyl group orientations are slightly different: in 1ILH.a it is involved in a H-bond with the S247, while in 4J5X this interaction is absent and the hydroxyl group is shifted toward the α10 helix. In the 1ILH.b structure the ligand orientation is opposite to the previous ones, with a phosphate group forming a H-bond with H407, and the hydroxyl group pointing toward the W299 residue. In 1NRL the ligand maintains the same interactions, with the addition of another H-bond between S247 and the second phosphate group. Finally, in 1ILH.c the phosphate groups establish H-bonds with S247 and Q285, while the hydroxyl group points toward the R410. The 3HVL structure^21^ presents a ligand orientation identical to that in 1NRL; for this reason, only the latter deposition, with better resolution and few missing residues, was retained for the analysis.

**Figure 2:**
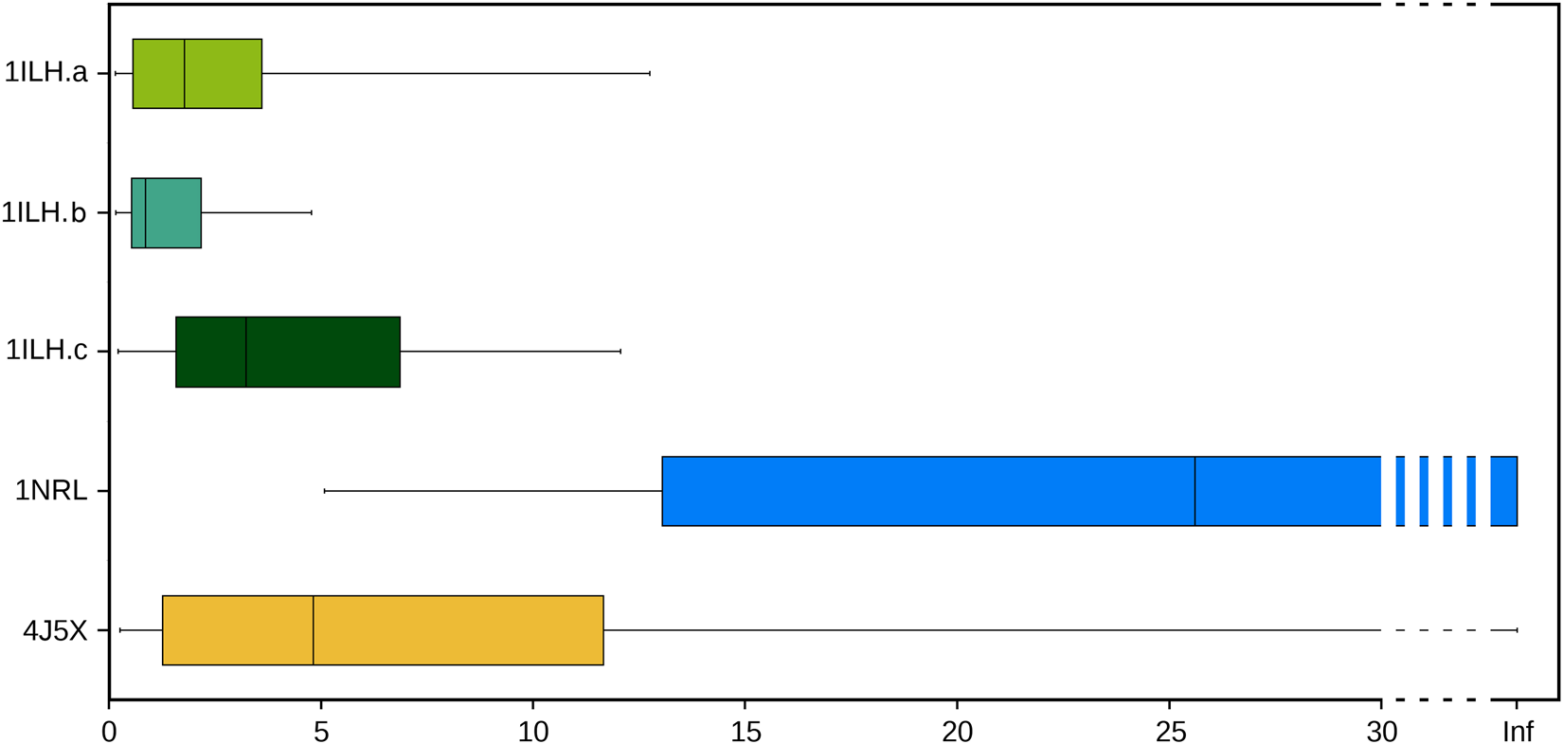
Boxplots showing the time necessary to leave the initial experimental ligand binding geometries, computed from all the SMD simulation replicas.

To gain insights into the characteristics of the available experimental ligand binding modes in PXR, we decided to investigate their relative stability using the SMD approach^37,38^. In SMD simulations, the potential energy of the system is scaled, thus lowering the energy barriers and facilitating the barrier-crossing events. This method was proposed for the study of the whole ligand unbinding process and the prediction of kinetics constant^39,40^, but has already been used to evaluate the stability of different binding poses^36,41^. We simulated the PXR-LBD in absence of protein partners starting from the five different experimental ligand orientations presented above. For each starting structure we generated 23 replicas of 30 ns and, for each of them, we evaluated the time necessary to reach a ligand RMSD value of 4 Å from the initial geometry. In Fig. 2, the boxplots represent the statistics obtained from the different replicas.

From this analysis, the binding mode in 1NRL resulted the most stable among the available experimental geometries, with a median value of 25.6 ns to leave the initial geometry, and 11 replicas that never approached the RMDS value of 4 Å. The second most stable geometry was that in 4J5X, with only one replica that maintained the initial conformation for the whole simulation and a median value of 4.8 ns to leave the initial geometry, far smaller than the 1NRL one. All the 1ILH structures were unstable and rapidly drifted from the starting geometry, indicating that these depositions may have not captured the most stable binding modes of the ligand. None of the performed replicas reached a fully solvated state, but only internal ligand reorientations have been observed in the simulation time.

### Prediction of the binding path

The PXR cavity is buried and does not presents any channel for solvent access. While the experimental crystal structures provide information about the final bound state of the ligands, no established experimental techniques are available for describing the dynamics of the ligand binding processes at an atomistic level. To computationally simulate this process and predict the path for ligand entrance into the cavity, here we employed the MD-Binding method^12^ that makes use of an additive external force to enhance sampling of the binding event.

**Figure 3:**
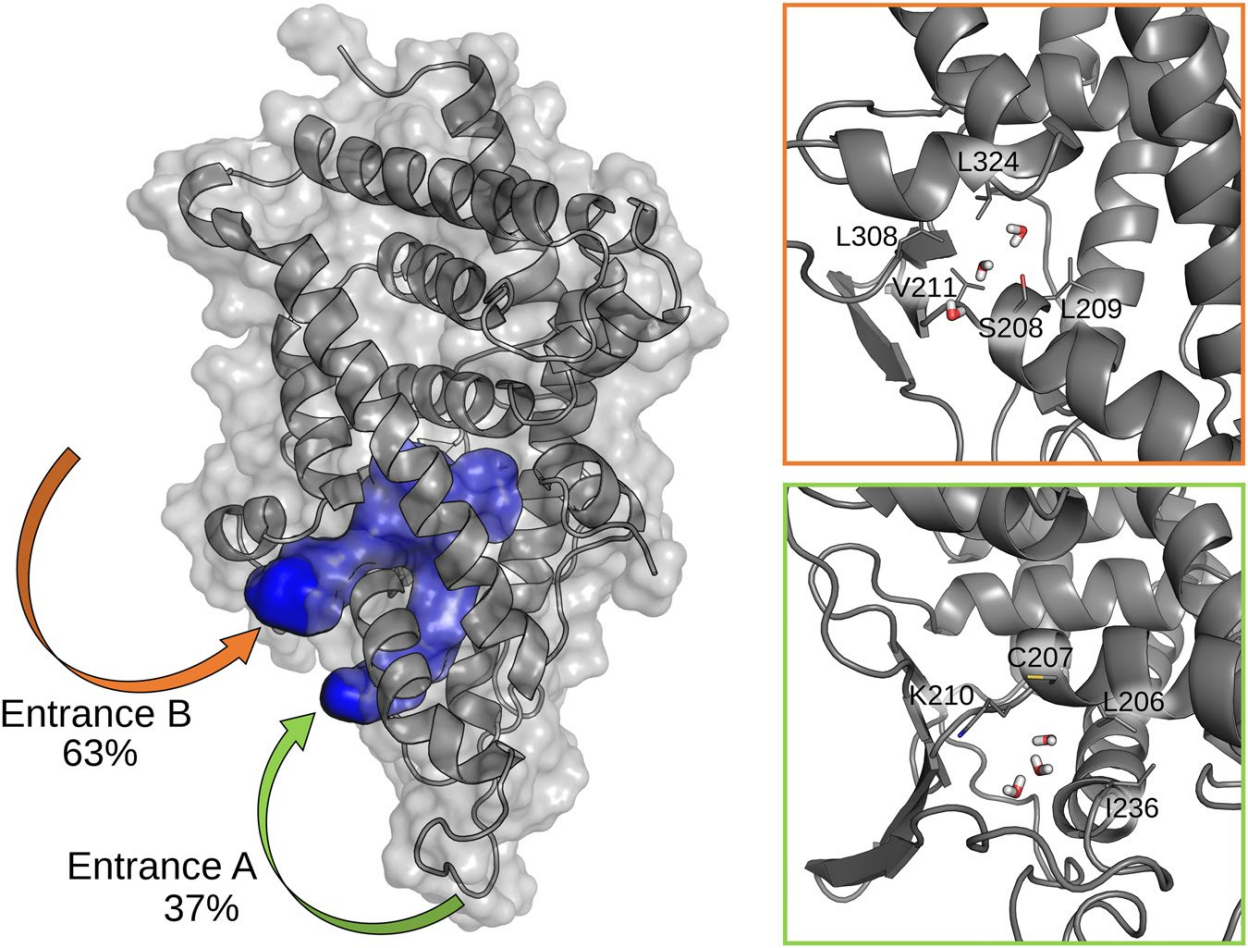
Alternative accesses of water molecules into the PXR cavity. On the left: the solvent-accessible volumes found during the simulation are represented as a solid blue surface; the two entrance channels are indicated by arrows. On the right: focus on the two entrances (top: B, bottom: A) with sidechains of the residues responsible for gate opening and water entrance represented as sticks.

The originally proposed protocol of MD-Binding would require characterization of the binding pocket through the Nanoshaper^42^ software, which identifies the atoms facing the pocket entrance in the protein structure. Due to the buried nature of the PXR cavity, this approach did not recognize any entrance channels in all the crystal structures. To overcome this limitation, we performed a plain MD simulation of the apo protein structure (PDB ID: 4J5W) in explicit solvent and analysed the water molecules exchanged between the bulk solvent and the binding cavity. During 100 ns simulation, about 300 water molecules were exchanged (entered or left), probably indicating the presence of a gate opening that allows for a fast water transition. Looking for the access points of these water molecules, we found a transient formation of two entrance channels connecting the bulk solvent with the binding region (Fig. 3).

We called entrance A the channel that was formed between α2 and α3 and entrance B that between α2 and α6. We observed that the residues of both the entrances are not involved in the interaction interfaces with RXR and SRC-1.

Despite during the simulation 63% of the water molecules were exchanged through entrance B, indicating a clear preference for this pathway, this finding could not be sufficient to establish the preferred entrance path for the ligand. Differences in size and physico-chemical properties between water and the ligand molecules may indeed change the energetic barriers associated to their entrance. For this reason, we started an MD-Binding campaign to investigate the SR12813 ligand binding through both the identified channels.

**Figure 4:**
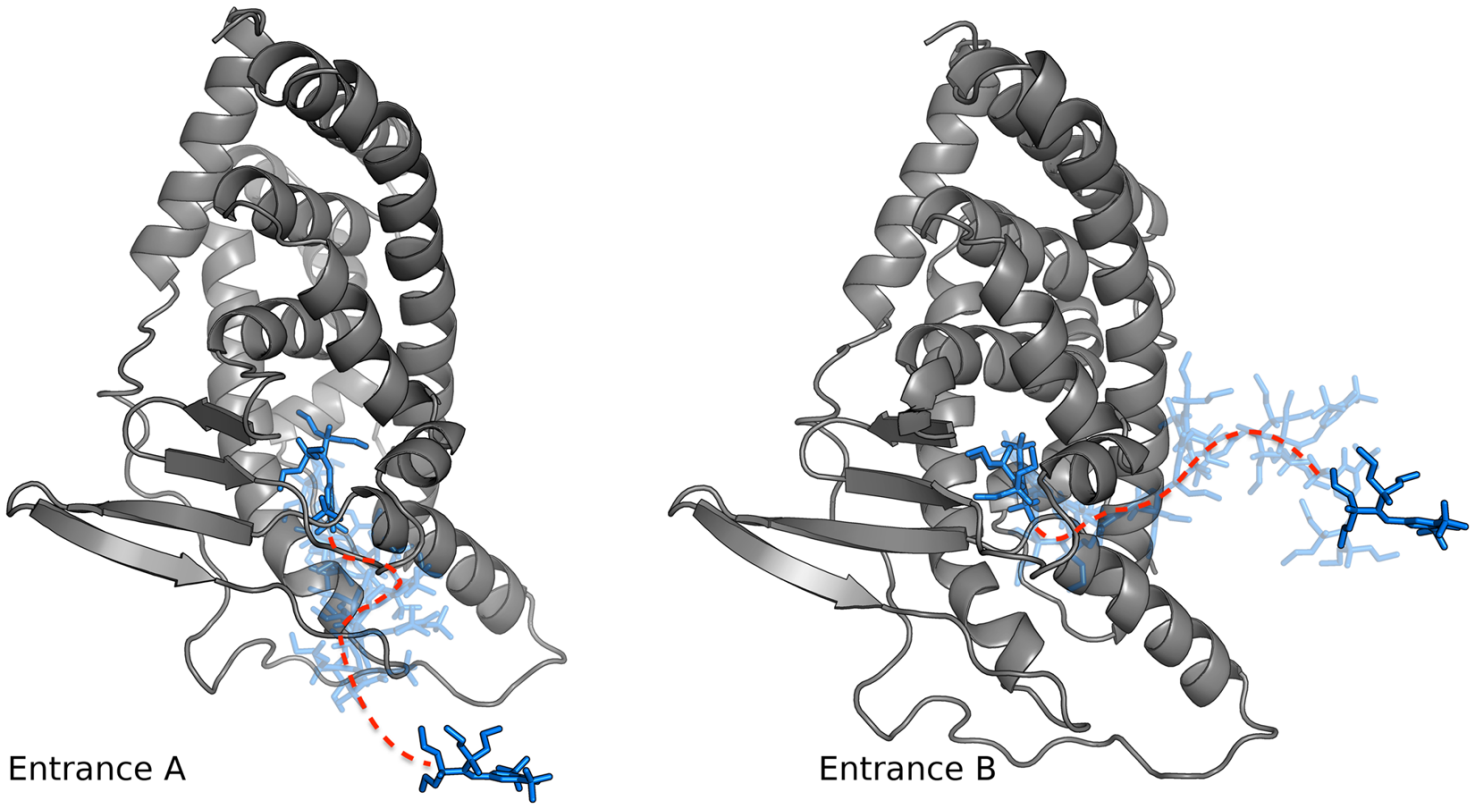
Ligand binding paths associated to the two entrances. Protein is represented as grey cartoons, the first and last frames in the ligand trajectory as solid blue sticks, and the trajectory as transparent sticks.

We first selected two frames from the simulation of the apo protein in which both channels A and B were opened and used Nanoshaper to identify the atoms at the entrance of the cavity. This information was then used by the BiKi software for the initial ligand positioning outside the binding cavity (point A, for entrance A, and B, for entrance B). We started 50 replicas of 20 ns for each entrance starting from the apo structure, thus collecting a total of 2 μs of simulation. Once the simulation campaign was completed, we pruned out the replicas ending without the ligand approach to the switch-off residue and analysed the remaining replicas. Only 8 simulations starting from point A, against the 42 starting from point B, reached the binding site. Despite the proximity of the two entrances, all simulations starting from point A passed through entrance A and those starting from point B through entrance B. Examples of the binding paths associated to the two entrances are shown in Fig. 4.

The results confirmed the preference of SR12813 for entrance B, with 84% of simulations overcoming the energetic barrier and reaching the buried binding site. An average time of 7.5 ns simulation was required to reach the cut-off distance from the switch-off residue, leaving more than 10 ns of plain MD for refinement of the ligand binding mode. Interestingly, we found that during most of the simulations of the path through the B entrance, the ligand causes a shift of the α6 helix (RMSD on Cα atoms of the α6 helix ranging from 4 to 8 Å), thus producing the disruption of a salt-bridge between the E321 and R410 residues (Supplementary Fig. S2). It was reported that the mutation of these residues alters the basal activity of PXR, highlighting their relevance in the binding process^12^. This conformational change was not found in the simulations of water entrance; therefore the salt-bridge acts as a gate for SR12813 and its breaking may be interpreted as the rate-determining step for the recognition process.

### Analysis of the predicted binding modes

Despite the high success rate in overcoming the energetic barrier for ligand binding obtained by MD-Binding simulations, the final geometries were highly heterogeneous. The distance RMSD (dRMSD, see Method section) values, calculated on the last frames (Supplementary Table S2) show that only few replicas reached a geometry similar to one of the five crystallographic structures. While only 1ILH.a was reproduced with high accuracy (dRMSD value for the replica B22: 1.1 Å) and acceptable results were obtained for 1ILH.b and 4J5X (dRMSD < 2 Å), none of the replicas approached 1ILH.c and 1NRL, despite the latter structure was predicted as the most stable one by SMD (Fig. 2). We verified that also over the whole MD-Binding simulation the ligand never approached these experimental binding modes. We attributed the difficulty of MD-Binding in reproducing the experimental structures to the buried nature of the PXR cavity that prevents the ligand free rotation upon entrance. Indeed, the plain MD simulation performed after the bias switching-off, only provided a local refinement. Even increasing the sampling time to 100 ns on four selected replicas was not sufficient to observe large conformational changes (Supplementary Fig. S3).

**Figure 5:**
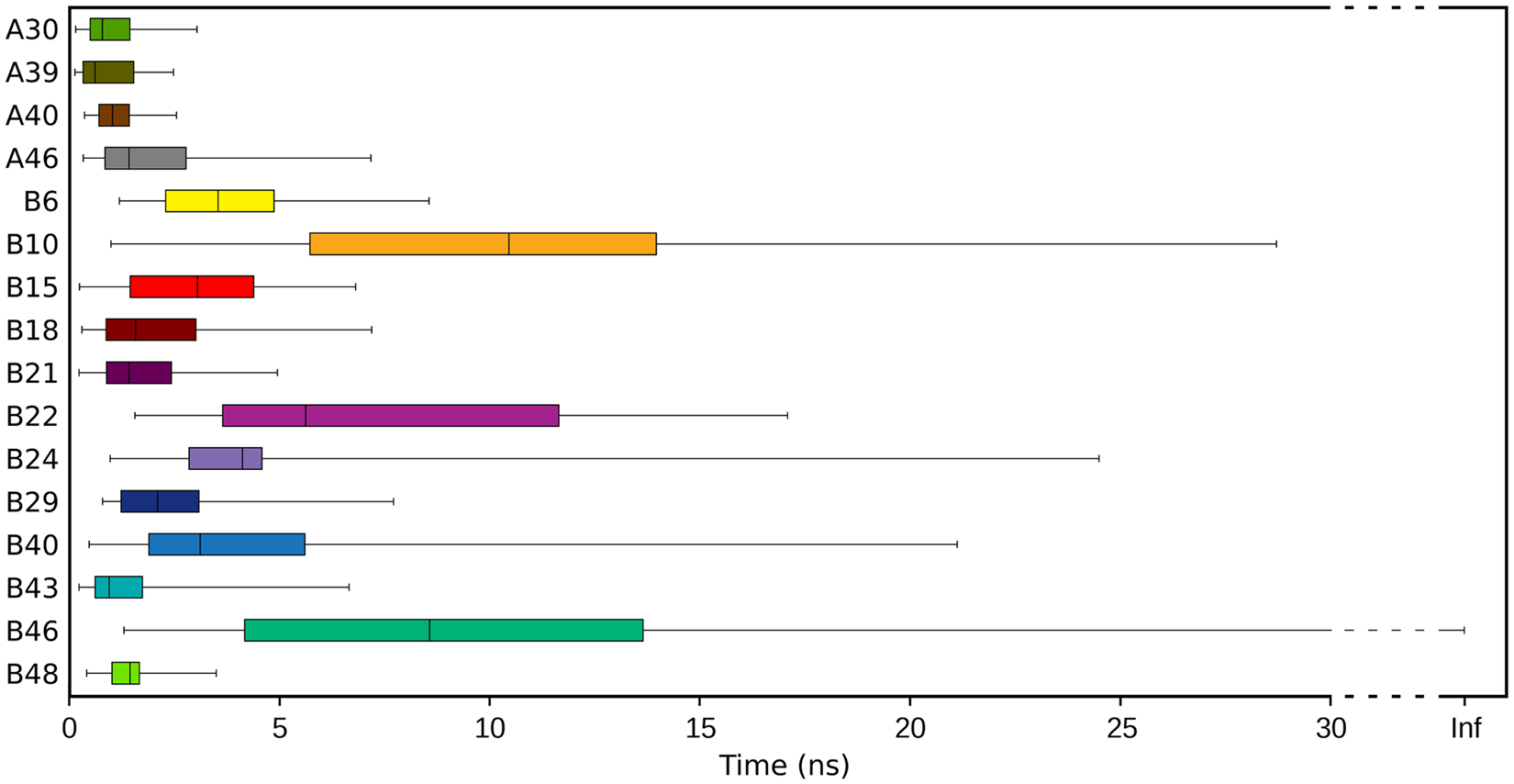
Boxplots showing the time necessary to leave the initial geometry of the MD-Binding poses, obtained using SMD simulations.

Therefore, to enhance sampling of the ligand conformational changes inside the binding cavity, we decided to perform SMD simulations, instead of plain MD, starting from the final frames of MD-Binding. We selected the frames in which the distance between ligand and the switch-off residue was smaller than 5.5 Å (obtaining 16 different starting points) and performed 20 replicas of 30 ns collecting a total of 9.6 μs of simulations. None of these starting binding geometries showed high stability and most of the simulations rapidly drifted away (Fig. 5; as a comparison, see the 1NRL stability in Fig. 2). Interestingly, one of the most stable replicas was the B22, that was very close to the 1ILH.a X-ray structure.

**Figure 6:**
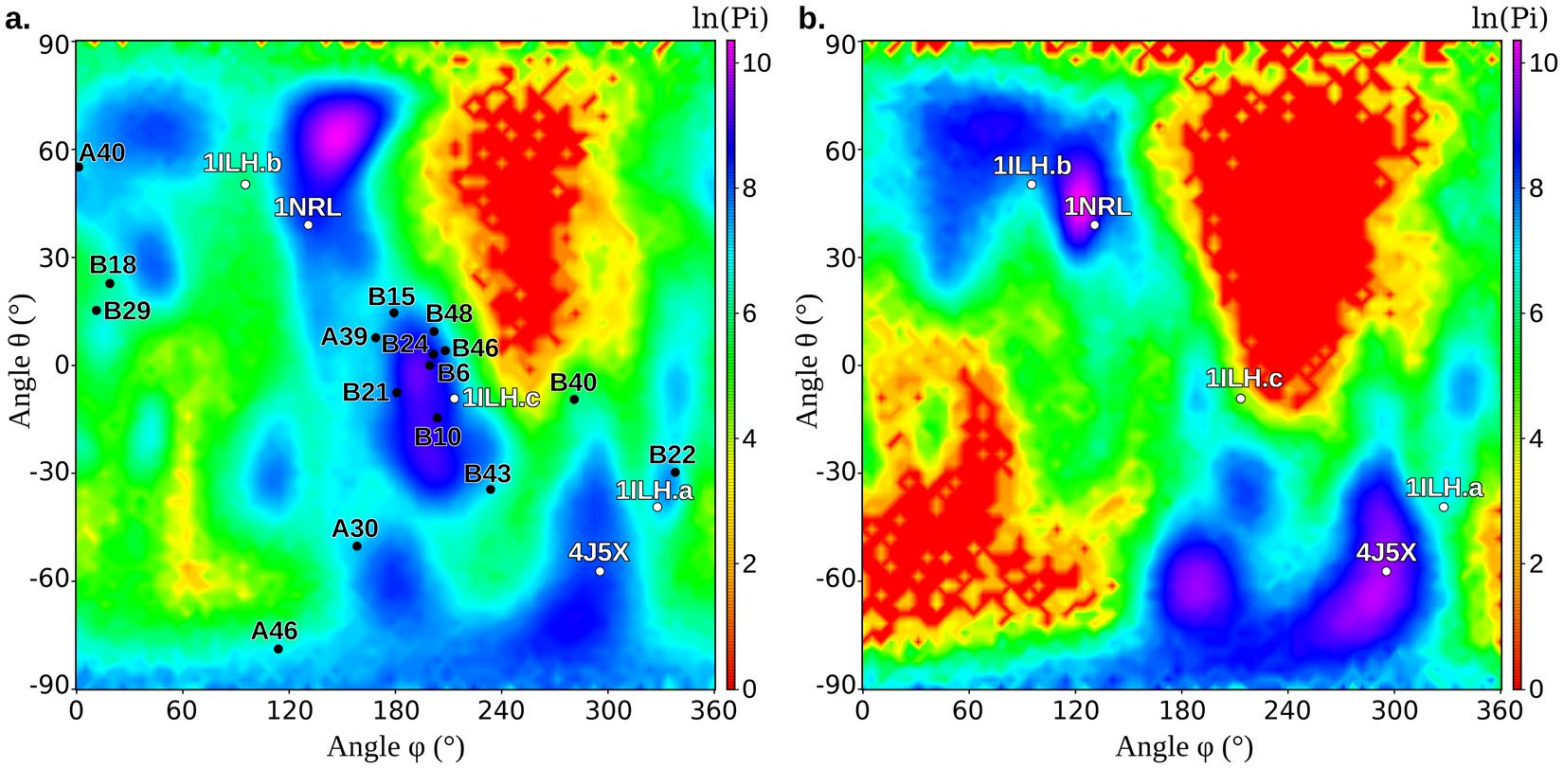
Conformational state probabilities obtained from the SMD simulations represented in the sub-space of the two CVs. Simulations: (**a**) starting from MD-Binding poses; (**b**) starting from the X-ray structures. X-ray geometries are projected onto the map as white dots, while the MD-Binding poses used as starting points for SMD simulations are represented as black dots.

To investigate the conformational space explored by the SMD simulations, we designed two CVs (θ and φ) that represent the ligand orientation within the cavity in polar coordinates (see Methods section). The conformational sub-space explored by the ligand during the SMD simulations is well represented by the probability distribution map depending on these variables, that highlights the most sampled regions (Fig. 6a).

This representation highlighted that most of the MD-Binding poses presented a ligand orientation similar to that in the X-ray 1ILH.c, with the phosphate groups pointing toward the F288 and S247 residues in the most interior region of the cavity, and the hydroxyl group pointing toward R410 at the B entrance. Moreover, it confirmed that the B22 simulation approached the 1ILH.a geometry, with the phosphate groups directed toward W299 and the hydroxyl group toward the αAF helix. The remaining MD-Binding poses were not close to any experimental structures. On the contrary, SMD mainly sampled the region nearby the 1NRL crystal structure (around θ = 60°, φ = 150°), despite in this zone no MD-Binding poses had been found. This implies that a number of SMD simulations drifted from the initial poses to reach the 1NRL region.

We also compared sampling performed by SMD starting from the MD-Binding poses with the one obtained starting from the X-ray structures (see the “Evaluation of experimental binding modes” sub-section), using the same two CVs (Fig. 6b). The similarity between the two maps indicates that the method well sampled the whole conformational sub-space. The main difference was observed in the region nearby 1ILH.c, that was highly sampled by SMD starting from the MD-Binding poses (Fig. 6a), while it was poorly sampled starting from any X-ray structures (Fig. 6b). This finding can however be explained considering the high number of MD-Binding poses falling in that region, representing similar ligand orientations. Another difference concerns the most sampled region in the two maps: while in SMD simulations starting from the X-ray structures it is close to 1NRL structure, in those starting from the MD-Binding poses it is shifted of about 20° toward higher θ values.

**Figure 7:**
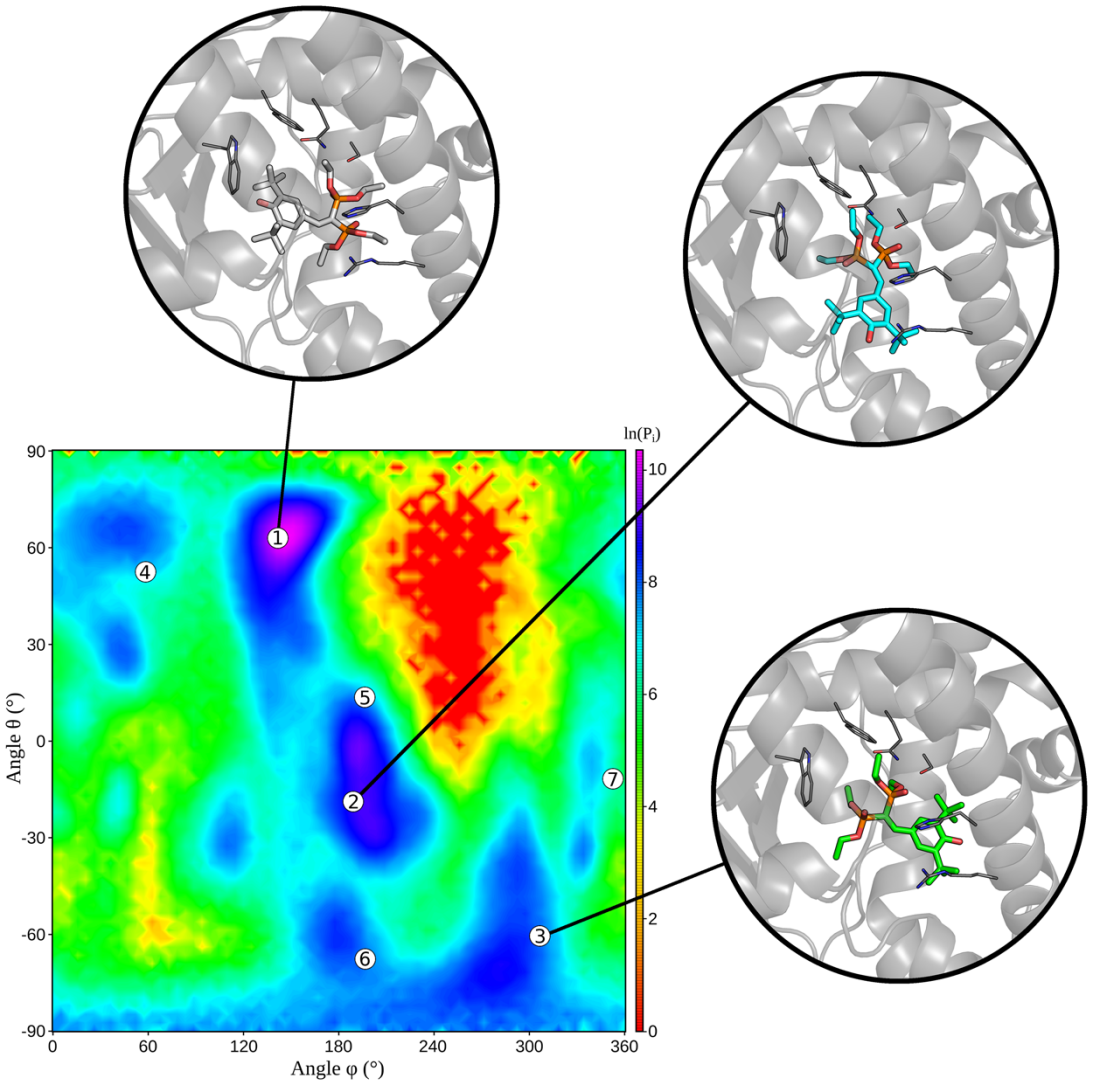
Centroids of the seven most populated clusters projected onto the conformational state probability surface obtained by SMD simulations starting from the MD-Binding poses. For the three most populated cluster, the 3D structure of the cluster centroid is also reported.

We extracted a limited number of poses from the whole ensemble representing the most sampled regions of the map using cluster analysis. The seven most populated clusters, out of the 227 obtained, well represent the zones with high conformational probability in the CVs subspace (Fig. 7).

In particular, the three most populated clusters contain ligand orientations similar to those observed in three experimental structures (1NRL, 1ILH.c, and 4J5X, respectively). Despite the dRMSD from the X-ray structures registered for the centroid of the most populated clusters were not very low (Supplementary Table S3), at least one conformation close to each of the X-ray ones (dRMSD <1.5 Å) was found within the whole set of cluster centroids (Supplementary Fig. S4).

## Discussion

Despite the extensive experimental information on the ligand-binding geometries in the PXR-LBD, some key features of the binding event have not yet been explained. Rationalization of the multiple binding sites and ligand orientations observed in the promiscuous binding cavity is the central issue. Moreover, mechanistic understanding of the binding process would clarify the mode of ligand entrance into the buried cavity. Elucidation of these aspects could be effectively exploited in drug discovery studies aimed at both understanding PXR activation by a wide range of chemicals and finding novel modulators of the PXR transcriptional activity^1,2^. Given the high flexibility of the LBD that is expected to characterize the binding process, these studies require advanced MD-based methods able to take into account the dynamics features of the whole ligand-receptor system, thus overcoming the limitations of the current molecular docking approaches.

Several promising enhanced sampling methods have been developed in recent years for computing ligand-receptor association and dissociation mechanisms^30,43^. These approaches were initially focused on simulating the association process, with the aim of accurately describing the mechanism and the thermodynamic features of the process^32–34,44^. More recently, evidences that drug efficacy well correlates not only with binding affinities but also with binding kinetics, as well as the increasing computational power, have stimulated the development of methods for the prediction of binding kinetics. Among them, metadynamics-based methods emerged for their ability to characterize the unbinding pathways, transition states and kinetic constants^45,46^. Most of the proposed methods make use of Markov State Model to build a kinetic network model^46–48^, others combine MD with Brownian dynamics and milestoning theory^49^, or apply random forces to the ligand to accelerate the unbinding event^50^. Within this framework, the BiKi Suite^35^ provides several tools to both investigate the binding pathways and obtain hints about the key determinants of the binding event. The choice of the most appropriate method depends on the specific challenges posed by the system under study and the required computational efforts vary with the method accuracy.

Here we investigated the binding mechanism of the most studied PXR ligand, SR12813. The intrinsic flexibility of the domain involved in ligand binding and the buried nature of the cavity, as well as the lack of information about the binding pathway, make this system a challenging task for all the above-mentioned methods. With the aim of obtaining mechanistic insights on this association process, we used the MD-Binding method to simulate the ligand entrance into the cavity and SMD simulations to extend sampling of the bound states. Our results provided clear indication that the combination of these methods can produce a complete picture of the binding event, ranging from the prediction of the binding path to the exploration of different putative binding modes. The challenging study-case led us to develop specific methodological solutions that could be useful also for the study of other systems. In the case of buried cavities, we suggested a preliminary exploration of the ligand entrance pathway through the analysis of water entrance. Moreover, to assess if MD-Binding have reached all the possible binding modes, we suggested the use of additional SMD simulations.

Overall, the obtained results provide several insights into the SR12813 binding mechanism. The ligand preferentially enters the binding cavity through the B entrance, between the α2 and α6 helices. A significant ligand-induced conformational change of the α6 helix, that causes the break of the E321-R410 salt-bridge, was observed in our simulations and was interpreted as the rate-determining step of the binding process. The initial arrangement of SR12813, with the phosphate groups oriented toward the interior of the cavity, directed the MD-Binding simulations toward poses similar to that observed in the 1ILH.c structure. We consider the 1ILH.c geometry as a relative minimum in the free energy surface associated to the ligand binding process, that is reached just upon the ligand entrance. In fact, we did not observe a high stability for this geometry, neither in the SMD simulations starting from the X-ray structures, nor in those starting from the MD-Binding poses. On the contrary, the most stable geometry resulted that of 1NRL, as indicated by both the SMD calculations performed, even if no MD-Binding simulation reached it. It is interesting to note that the ligand orientation observed in the 1NRL structure was the only one found in two PDB depositions (1NRL and 3HVL), obtained from two different experimental groups.

It was proposed that the two protein partners that were co-crystallized with PXR in some of the available structures might have played a role in the stabilization of a particular conformation of the SR12813-PXR complex^20^. In fact, the similar 1NRL and 3HVL binding geometry were found in presence of SRC-1, the 4J5X geometry corresponds to PXR bound to both SRC-1 and RXR, while the most miscellaneous deposition 1ILH, that depicts a more flexible image of the SR12813 binding, is associated to the PXR LBD crystallized without protein partners.

Our simulations were based on PXR LBD structures with no partners, and accordingly they described the ligand binding event as characterized by high flexibility and plasticity of the protein domain. Our results provide further insights in addition to the hypotheses based on experimental data. In fact, they support the presence of multiple pre-existing conformational states of the SR12813-bound LBD also in absence of the protein partners. This could be considered as a particular case of conformational-selection, in which RXR or SRC-1 can select one among these different metastable states of the complex thus shifting the dynamic population equilibrium toward a specific bound state. The analysis of the multiple accessible states detected in our simulations can explain the different binding modes observed by X-ray crystallography for the SR12813 ligand and support the hypothesis that the 1ILH binding geometries are kinetically favoured states while 1NRL could represent the most thermodynamically favoured state. Future studies based on rigorous approaches for computing both thermodynamic and kinetic properties^46,48^ could improve understanding of the role of the above described multiple states in the binding/unbinding processes.

The approaches here proposed for studying ligand binding to PXR effectively treated the dynamics of this system during binding and shed light on some of the unresolved mechanistic issues. On the basis of this positive outcome, these methods appear suitable for analysing the mechanistic features of other ligand binding processes involving promiscuous protein domains.

## Methods

### System preparation

Crystal structures for PXR in its unbound (PDB ID: 4J5W^8^) and SR12813-bound (PDB ID: 1ILH^12^, 1NRL^20^, 4J5X^8^) forms were obtained from the Protein Data Bank^51^ (PDB), and protein partners were removed. The PXR structures have unresolved regions between the α1 and α2 helices, that was modelled using Prime^52^ within the Maestro Schrodinger Suite. Proteins were prepared with the Protein Preparation Wizard^53^ included in Maestro: hydrogen atoms were added, all water molecules removed, C and N terminal capping were added, disulphide bonds were assigned, and residue protonation states were determined by PROPKA^54^ at pH = 7.0. The SR12813 ligand was parametrized using the BiKi suite^35^ with the AM1-BCC^55^ level of theory. Partial charges were derived using the RESP method^56^ in Antechamber^57^, while a GAFF^58^ parametrization was used to achieve the complete topological description of each ligand. Sensible torsion parametrization of the C=C-Ca-Ca angle was compared with QM calculations performed at the HF/6–31G* level using the Jaguar^59^ program in Maestro to adjust the ambiguous parametrization of GAFF.

### Plain MD simulation

The plain MD simulations were performed using GROMACS 4.3^60^. The protein was solvated in an orthorhombic box with TIP3P water molecules^61^, ad neutralized with Na^+^/Cl^−^ ions within BiKi basics^35^. The minimal distance between the protein and the box boundaries was set to 12 Å. The Amber ff14SB force field^62^ was used for the proteins and a multistage equilibration protocol was applied to remove unfavourable contacts and provide a reliable starting point for the production runs: the system first underwent 5,000 steps of steepest descent energy minimization, and then four different consecutive equilibration steps were performed: (1) 100 ps in NVT ensemble at 100 K using a time-step of 1 fs, (2) 100 ps in NVT ensemble at 200 K and time-step increased to 2 fs, (3) 100 ps in NVT ensemble at 300 K, and (4) 1,000 ps in NPT ensemble at 300 K. In all the stages the atoms belonging to the protein backbone (and to the ligand, where present) were restrained with a force constant of 1,000 kJ mol^−1^ nm^−2^. Electrostatics was treated with the cut-off method for short-range interactions and with the particle mesh Ewald method^63^ for long-range interactions (rlist = 1.1 nm, cut-off distance = 1.1 nm, vdW distance = 1.1 nm, PME order = 4). The constant temperature conditions were provided using the velocity rescale thermostat^64^ (coupling constant of 0.1 ps), while pressure was coupled with the Parrinello-Rahman barostat^65^ (coupling constant of 2 ps). All bonds were constrained with the LINCS algorithm^66^.

### MD-Binding simulations

The MD-Binding method^36^ within the BiKi suite^35^ uses an additive external force that is summed to the regular potential energy of the system to enhance the probability to observe the binding event. The bias consists in external electrostatic-like forces acting between a subset of the residues of the binding site and the ligand. The intensity of the biasing force is regulated by the adaptivity rules and gradually switches off as the process moves forward so that, after the conjectured passing of the transition state has occurred, it slowly recovers the behaviour of classical unbiased MD.

In the standard protocol for MD-Binding, the possible entrances for the ligand are computed using Nanoshaper^42^, and the ligand is positioned with a random orientation at a predetermined distance from the residues that form the entrance. In the present work, we used two selected frames extracted from the apo simulation, to calculate the entrance with Nanoshaper for the ligand positioning. The protein attractive atoms were selected as the list of residues that belong to the internal cavity of PXR according to Nanoshaper calculations (C207, S208, L209, K210, V211, L239, H242, M243, M246, S247, F251, F281, C284, Q285, F288, W289, Y306, L308, E309, T311, A312, G313, L318, E321, M323, L324, F326, H327, I403, H407, R410, L411, F420, M425, F429); for the ligand, we used all the heavy atoms. As switch-off residue we selected the S247, with a cut-off distance of 4 Å. This residue was found to interact with the ligand in most of the experimental structures and is placed at the opposite side of both the entrances so that, when the ligand approaches its atoms, the transition state for the binding process could be considered overcome. 50 independent simulation runs were launched in parallel per entrance gate, each of them during 20 ns, starting from the 4J5W apo protein conformation.

### Scaled MD

In the SMD approach implemented in BiKi^40^, the potential energy terms are scaled by a scaling factor λ to reduce the energetic barrier and enhance sampling during the simulations. In the present work we used the value λ = 0.5 and applied low restraints (50 kJ mol^−1^ nm^−2^) to the protein backbone atoms, excluding the residues around the binding site (within 8 Å from the ligand) that were kept unrestrained. Simulations of 30 ns were carried out for each replica in the NVT ensemble.

### Analysis of MD simulations

For the comparison of binding modes, we calculated the dRMSD between the ligand-site distances in the simulated complex and the corresponding ligand-site distances in the X-ray structures:

$${\rm{dRMSD}}=\sqrt{\frac{{\sum }_{i}{\sum }_{j}{({d}_{ij}^{x}-{d}_{ij}^{m})}^{2}}{N}}$$

where x and m are the experimental and calculated complexes, respectively; d are the vectors of the distances between the ligand and the binding-site heavy atoms; i and j are the indices of the atoms; and N is the number of comparisons performed. We defined all the protein atoms within 6 Å from the ligand heavy atoms as binding-site heavy atoms. Calculations were performed with PLUMED^67^. Using the dRMSD index, the distance calculation takes into account only the deviation of the relative position of the ligand with respect to the residues belonging to the binding site; it is a better index to evaluate the accuracy of the binding geometry than the RMSD calculated on the absolute positions of the ligand atoms, which neglects the difference in the positions of protein residues in the simulated and reference geometries.

To evaluate the stability of a ligand binding conformation with SMD, we measured the time required for the ligand to reach 4 Å of RMSD (computed on heavy atoms) from the initial geometry in each replica, and we evaluated the resulting boxplot. The cut-off value of 4 Å was chosen analysing different RMSD graphs to assure that it was a good cut-off value to discriminate when the ligand definitively left its starting conformation.

**Figure 8:**
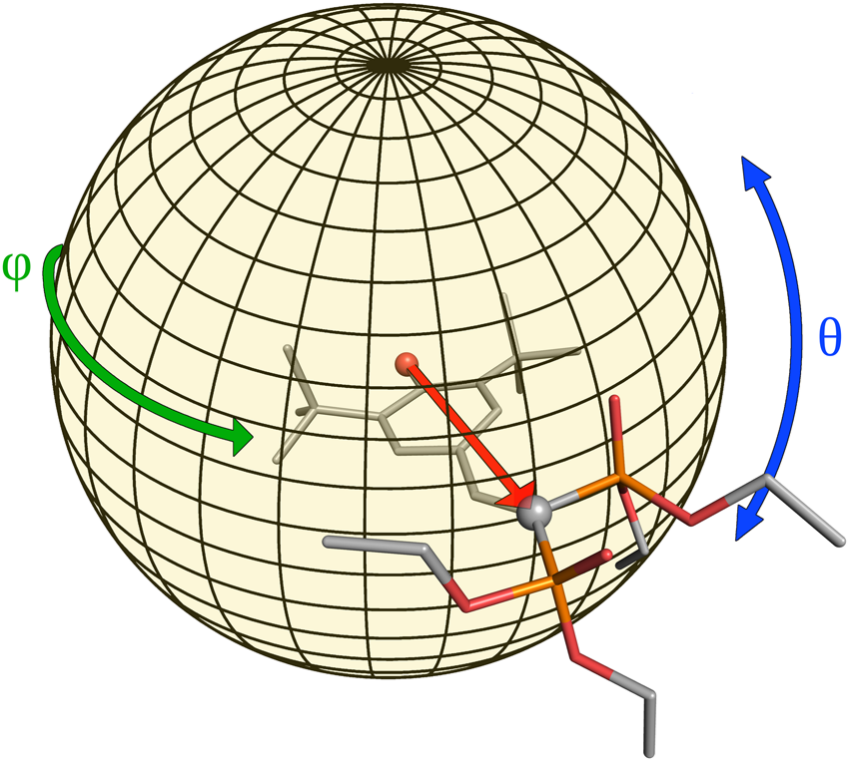
Representation of the CVs describing the ligand orientation inside the cavity. The angles θ and φ are computed for the vector connecting the oxygen of the hydroxyl group of the ligand and the carbon atoms between the two phosphate groups.

The ligand orientation within the binding site were represented in the sub-space of two selected collective variables (CVs) that correspond to the polar coordinates θ and φ in a reference system centred in the oxygen atom of the hydroxyl group of the ligand, relative to the vector connecting this atom and the carbon atom joining the two phosphate groups (Fig. 8). These coordinates were computed for each conformation in the trajectory, upon alignment of the protein Cα atoms.

In this way we correctly described the ligand rotation inside the cavity, neglecting the possible translations. The conformational sampling performed in each simulation was then represented in the sub-space described by these two coordinates as a probability density surface in which the counting of each bin was reweighted to account for the different dimensions of the bins due to the spherical shape of the surface.

After structural alignment on protein Cα atoms, ligand conformations sampled during the SMD simulations starting from the final MD-Binding geometries were clustered using the GROMOS RMSD-based clustering tool applied to the ligand heavy atoms, using a cut-off value of 2 Å.

## Electronic supplementary material

Supplementary Information

## Data Availability

All data generated during the current study are available from the corresponding author on reasonable request.
