# Supplementary material for: Exploring the PXR ligand binding mechanism with advanced Molecular Dynamics methods
Source: Sci Rep. 2018 Nov 1;8:16207. doi: 10.1038/s41598-018-34373-z (PMC6212460; doi:10.1038/s41598-018-34373-z)
Supplement: Supplementary file 1 — Supplementary Information [file 41598_2018_34373_MOESM1_ESM.pdf]

Exploring the PXR ligand binding mechanism with advanced Molecular Dynamics methods

Stefano Motta, Lara Callea, Sara Giani Tagliabue, Laura Bonati*

Table S1: Experimental structures for the PXR LBD in complex with SR12813 available in the PDB.

PDB ID	resolution [Å]	co-crystallized partner	ref	residues within 4 Å of SR12813
1ILH	2.8	none	12	**1ILH.a** S208, V211, M243, M246, S247, F251, F281, C284, Q285, F288, W299, L411<br>**1ILH.b** E205, L206, L209, L240, S247, F288, W299, M323, L324, H407, R410, L411, F420<br>**1ILH.c** L209, M243, A244, S247, F251, F281, C284, Q285, F288, M323, H407, R410
1NRL	2.0	SRC-1	20	L209, M243, A244, M246, S247, F251, F281, Q285, F288, W299, Y306, M323, H407, L411, F429
3HVL	2.1	SRC-1	21	L209, M243, A244, M246, S247, F251, F281, Q285, F288, W299, Y306, M323, L324, H327, H407, L411, I414, F420, M425, F429
4J5X	2.8	SRC-1 and RXR	8	L206, L209, L240, M243, M246, S247, Q285, F288, W299, Y306, M323, H327, H407, R410, L411, I414, M425

Table S2: dRMSD from the X-ray structures calculated on the last frames of the MD-binding simulations.

**dRMSD (Å) Entrance A**					
**Rep**	**1ILH.a**	**1ILH.b**	**1ILH.c**	**1NRL**	**4J5X**
A26	9.70	6.57	8.35	7.01	8.52
A30	7.04	5.85	5.44	6.23	4.97
A39	7.89	6.92	5.61	7.21	5.78
A40	4.62	3.46	5.13	3.74	4.57
A46	4.00	5.17	4.74	5.22	2.78
A47	5.32	4.83	5.53	5.48	4.58
A48	5.88	5.41	5.35	6.01	4.37
A50	5.18	5.54	5.69	5.29	4.90
**dRMSD (Å) Entrance B**					
**Rep**	**1ILH.a**	**1ILH.b**	**1ILH.c**	**1NRL**	**4J5X**
B1	4.72	4.15	5.18	4.55	4.95
B2	5.40	2.25	5.52	3.64	6.14
B4	8.45	4.68	5.31	5.35	6.64
B5	8.96	4.50	5.80	5.35	7.65
B6	5.88	3.15	2.56	3.93	4.79
B7	10.50	5.82	6.59	6.98	8.45
B9	9.70	6.73	5.74	8.48	7.42
B10	5.76	4.27	2.49	4.79	3.42
B11	5.63	3.72	5.74	4.75	5.48

B12	8.81	4.77	5.64	5.79	7.44
B14	8.86	4.85	5.70	5.71	7.57
B15	6.22	3.24	4.24	3.43	5.99
B16	4.70	4.44	4.58	4.81	4.01
B17	7.77	4.48	3.80	6.09	5.67
B18	2.51	1.92	4.57	2.75	3.63
B19	4.13	3.84	5.39	3.99	4.62
B21	6.46	3.81	3.64	4.08	5.37
B22	1.10	4.59	3.51	4.46	1.88
B23	6.06	4.13	4.86	6.16	5.81
B24	5.91	3.00	2.40	3.95	4.54
B26	5.52	5.11	3.43	6.25	4.54
B27	6.25	5.90	5.58	6.50	6.12
B28	8.09	4.65	4.19	5.87	6.32
B29	3.42	2.63	4.84	2.99	4.15
B30	8.75	5.08	5.02	6.12	6.90
B31	6.33	3.94	3.10	5.21	5.38
B32	8.87	6.81	5.31	8.28	6.36
B33	4.41	3.13	5.16	4.24	4.67
B34	5.05	3.74	5.10	4.74	4.95
B35	4.94	3.89	5.28	4.65	5.02
B36	4.94	4.82	5.16	4.82	5.80
B37	7.86	4.70	3.90	6.04	6.28
B38	5.07	2.99	5.02	4.45	4.81
B39	5.17	4.81	5.75	5.13	5.16
B40	3.27	3.64	2.44	3.81	2.49
B41	7.09	3.45	6.31	5.75	6.34
B43	4.61	4.15	2.72	4.89	2.93
B44	5.00	3.20	5.27	4.63	4.58
B46	5.05	2.95	2.19	3.84	4.21
B47	8.09	4.09	4.17	5.30	6.11
B48	5.41	2.74	2.28	3.41	4.26
B50	4.63	4.03	2.47	5.10	2.62

Table S3: dRMSD from the X-ray structures calculated for the centroids of the seven most populated clusters. The lowest distance from the X-ray structures registered for each cluster is highlighted in bold.

dRMSD (Å)					
**CLUSTERS**	**1ILH.a**	**1ILH.b**	**1ILH.c**	**1NRL**	**4J5X**
1	5.09	**1.97**	3.68	2.42	4.92
2	5.62	3.88	**2.19**	4.60	3.18
3	2.74	4.53	3.37	4.67	**1.85**
4	3.72	**2.00**	4.77	2.22	4.55
5	5.22	2.66	**2.44**	3.21	3.75
6	4.53	4.68	3.00	4.86	**2.92**
7	**1.36**	3.96	3.61	4.08	2.11

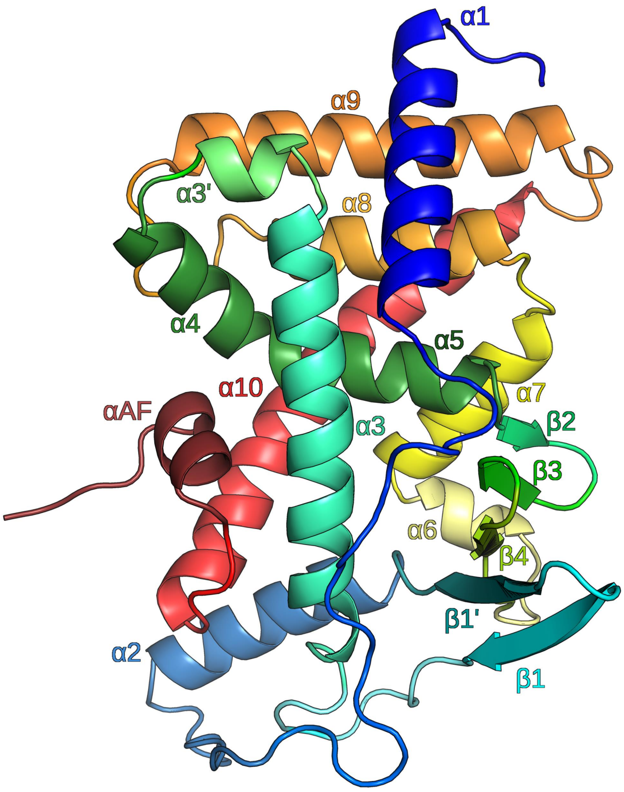

Figure S1: PXR structure in its apo form (PDBID: 4J5W) with secondary structure element names labelled according to the NR LBD nomenclature.[12]

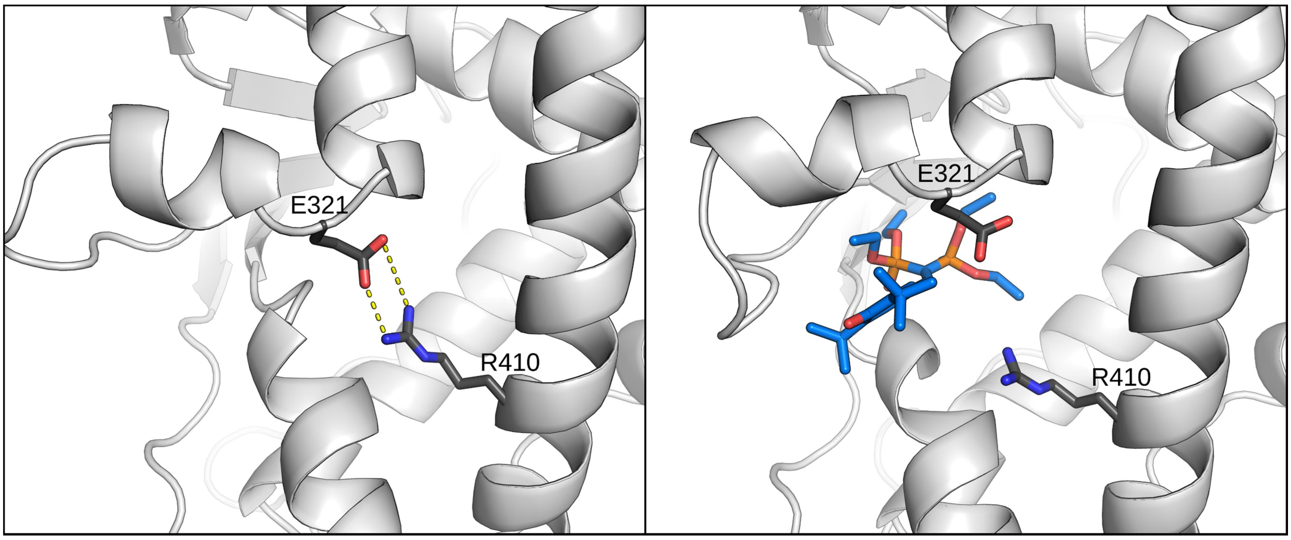

Figure S2: Breaking of the salt-bridge between E321 and R410 upon the ligand entrance. The unperturbed state (left) and a frame captured during the ligand association path (right) are reported. Protein is shown in gray cartoons, sidechains of residues forming the salt-bridge in dark grey sticks and ligand in blue sticks.

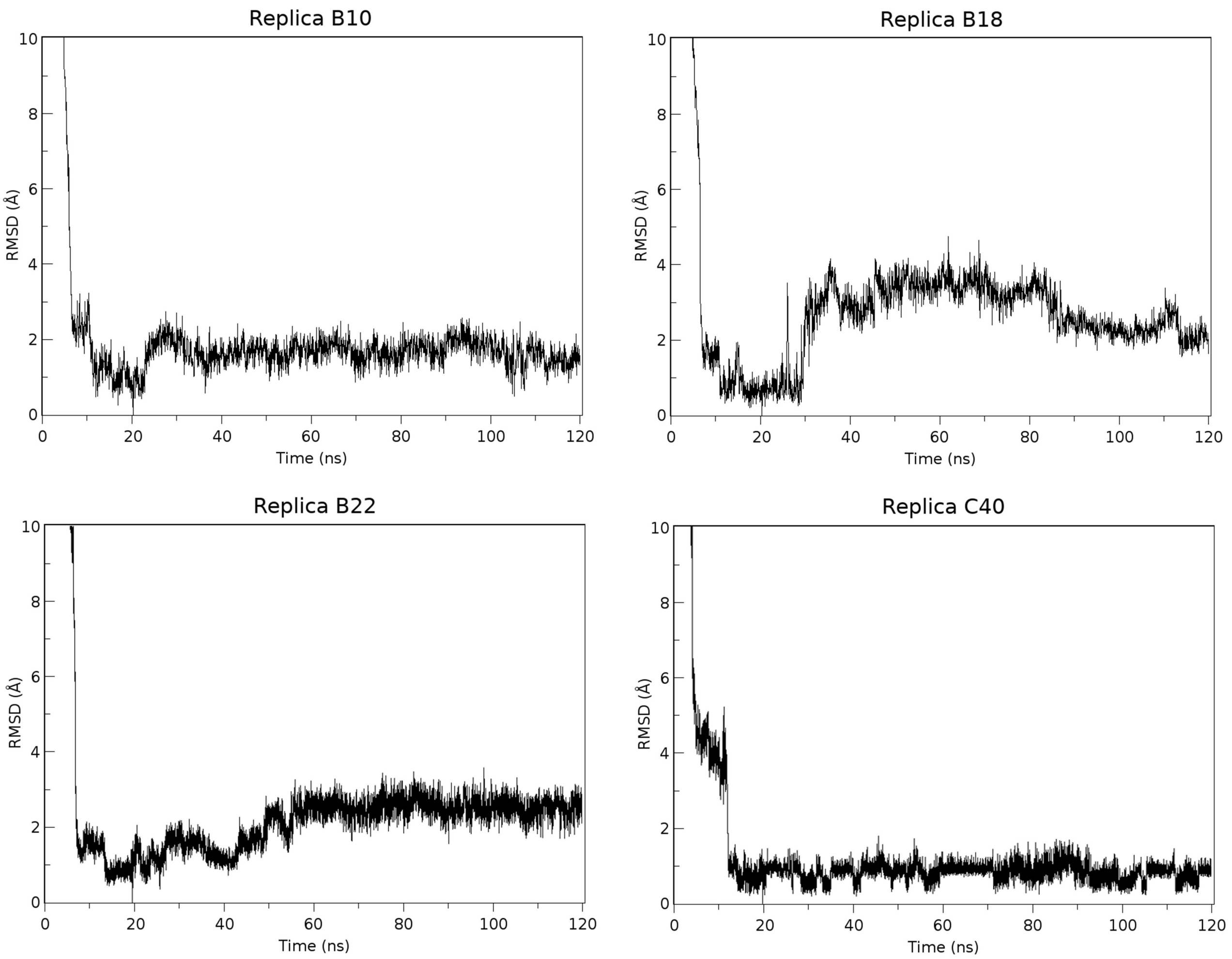

Figure S3: Graphs of the ligand RMSD from the MD-binding poses (calculated on heavy atoms) for MD-Binding simulations (0-20 ns) and plain MD restarts (20-120 ns).

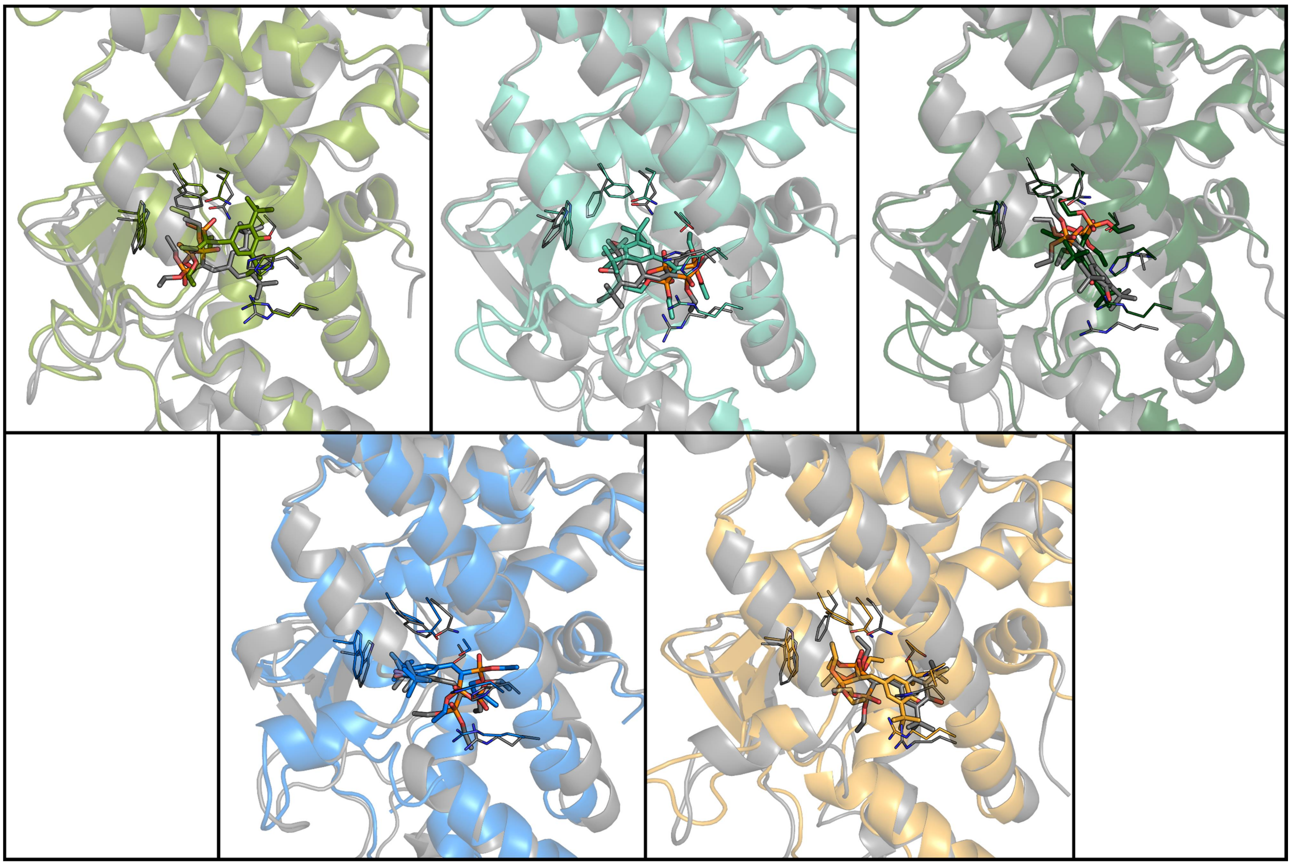

Figure S4: The cluster centroids nearest to the five X-ray structures. Cluster 46 is similar to 1ILH.a (dRMSD = 1.32 Å, light green); cluster 105 is similar to 1ILH.b (dRMSD = 1.42 Å, sea green); cluster 11 is similar to 1ILH.c (dRMSD = 1.52 Å, dark green); cluster 40 is similar to 1NRL (dRMSD = 1.43 Å, blue marine); cluster 41 is similar to 4J5X (dRMSD = 1.22 Å, bright orange).
